# Using Magnetic Resonance Imaging and Diffusion Tensor Imaging to Assess Brain Damage in Alcoholics

**Published:** 2003

**Authors:** Margaret Rosenbloom, Edith V. Sullivan, Adolf Pfefferbaum

**Affiliations:** Margaret Rosenbloom, M.A., is a research associate; Edith Sullivan, Ph.D., is a professor, and Adolf Pfefferbaum, M.D., is a professor, all in the Department of Psychiatry and Behavioral Sciences, Stanford University School of Medicine, Stanford, California. Margaret Rosenbloom also is a consultant and Adolf Pfefferbaum is director at the Neuroscience Program, SRI International, Menlo Park, California

**Keywords:** AODR (alcohol and other drug related) structural brain damage, magnetic resonance imaging, diffusion tensor imaging, brain imaging, patient assessment, neural tissue, gender differences, chronic AODE (alcohol and other drug effects), AOD dependent

## Abstract

Brain imaging using conventional magnetic resonance imaging (MRI) has revealed that several brain structures in people with a history of chronic alcohol dependence are smaller in volume than the same brain structures in nonalcoholic control subjects. Areas that are particularly affected are the frontal lobes, which are involved in reasoning, judgment, and problem solving. Older people are especially vulnerable to the damaging effects of alcohol. It is unclear whether women show consistently more vulnerability to these changes in the brain than men do. In general, alcoholics evaluated before and after a period of abstinence show some recovery of tissue volume, whereas alcoholics evaluated again after continued drinking show further reductions in brain tissue volume. A new MR technique called diffusion tensor imaging (DTI) can aid in detecting the degradation of fibers (i.e., white matter) that carry information between brain cells (i.e., gray matter). With DTI, researchers studying alcoholics have been able to detect abnormalities in white matter not visible with conventional MRI. Ultimately DTI may be useful in elucidating the mechanisms that underlie macrostructural and functional brain changes seen with abstinence and relapse.

Excessive chronic alcohol consumption is associated with significant shrinkage of brain tissue, degradation of fibers (i.e., white matter) that carry information between brain cells (i.e., gray matter), reduced viability of these brain cells, and impairment of associated cognitive and motor functions (for reviews, see Oscar-Berman 2000; Sullivan 2000). Some alcoholism-related tissue damage is partially reversible with abstinence, although residual tissue volume deficits persist even in long-abstinent alcoholics (Pfefferbaum et al. 1998).

Abnormalities are found in both the gray matter and the white matter of the brain. The brain’s gray matter consists of nerve cells (i.e., neurons), which account for its grayish color, and the surrounding glia cells, which provide mechanical support, guidance, nutrients, and other substances to the neurons. White matter is made up of long, thin extensions of the neurons, called axons, which carry information between neurons*.* White matter is paler in color than gray matter because the axons are surrounded by myelin––a fatty substance that protects the nerve fibers. The axons form fiber tracts linking nearby and distant neurons across different brain regions (i.e., white-matter tracts).

Imaging in living patients (i.e., in vivo) can be used to detect and quantify gray-and white-matter abnormalities on both macrostructural and microstructural levels. Conventional structural magnetic resonance imaging (MRI) reveals the size, shape, and tissue composition (gray vs. white matter) of the brain and its constituent parts. Diffusion tensor imaging (DTI) reveals the integrity of white-matter tracts that link regions of the brain to each other.

MRI is based on the observation that the protons of hydrogen atoms, when placed in a strong magnetic field, can be detected by manipulating the magnetic field. Because the human body is composed primarily of fat and water, it is made up mostly of hydrogen atoms. Variations in behavior of hydrogen atoms in different brain tissue types and structures show up as intensity differences that clinical structural MRI can detect and map to visualize and measure gross brain neuroanatomy.

Diffusion tensor imaging makes use of the fact that water molecules in the brain are always moving—that is, they are in Brownian motion. DTI detects the diffusion, or Brownian movement, of water protons within and between individual cells and yields measures of the magnitude and predominant orientation of this movement. The diffusion properties of water molecules within and between the three-dimensional elements, called voxels, that make up an image reveal the orientation and coherence (i.e., extent to which fibers align together) of fibers making up white-matter tracts.

Both MRI and DTI have been applied to the study of alcoholism. Structural MRI has been used for more than a decade to detect gross structural changes, such as tissue shrinkage and its reversal, and has identified brain regions that are particularly vulnerable to the toxic effects of chronic alcohol consumption. DTI, the more recently developed technique, is beginning to reveal microstructural abnormalities in white matter that are consistent with post mortem observations of white-matter damage, such as myelin loss, enlargement of microtubules (small tubular structures found inside nearly all cells), and degradation of membranes, even when that white-matter region appears normal on structural MRI.

## Structural MRI

Structural MRI studies of patients with chronic alcoholism are generally consistent with the literature on neuropathology and typically reveal reduced volume of both gray matter and white matter in the cerebral cortex, the folded outer layer of the brain. Older alcoholics show greater volume deficits relative to age-matched control subjects than younger alcoholics, suggesting that the older brain is more vulnerable to the effects of alcohol (Pfefferbaum et al. 1992). MRI usually shows that the greatest loss occurs in the frontal lobes, which are used in reasoning, working memory, and problem solving (Pfefferbaum et al. 1997). Changes also appear in other structures involved in memory, such as the hippocampus, mamillary bodies, thalamus, and cerebellar cortex (for a review, see Sullivan 2000).

Alcoholic men (Pfefferbaum et al. 1996) and women (Hommer et al. 1996) also show thinning of the corpus callosum, a band of white-matter fibers linking the brain’s hemispheres. Reduced volume also is reported in the pons, a largely white-matter structure of the brain stem that forms a critical node in multiple circuits linking the cerebellum—which regulates balance, posture, movement, and muscle coordination— to cortical regions of the brain involved in motor and sensory processing, as well as regions where these inputs are integrated (Sullivan et al. 2003). Alcoholics, particularly those with a history of seizures, show reduced white-matter volume in the temporal lobes (Sullivan et al. 1996). Alcoholics also have reduced white-matter volume in a part of the cerebellum known as the cerebellar vermis, where the loss is associated with deficits in postural stability (Sullivan et al. 2000). Lastly, chronic alcohol consumption can lead to specific neurological disorders involving white matter, such as Marchiafava–Bignami disease and central pontine myelinolysis (Charness 1993).

Certain structural MR images of alcoholics show areas of greater brightness in white matter, called white-matter hyperintensities (WMHIs) (Jernigan et al. 1991). These WMHIs can reflect a variety of underlying processes—including swelling caused by excess fluid (i.e., edema), the removal of the myelin sheath (i.e., demyelination), excess cell growth (i.e., gliosis), and increased extracellular fluid—some of which may eventually be documented and elucidated using DTI.

Long-term MRI studies of alcoholics in recovery or relapse have identified cortical white-matter volume as particularly amenable to recovery with abstinence (Shear et al. 1994) or vulnerable to further decline with continued drinking (Pfefferbaum et al. 1995; Pfefferbaum et al. 1998). How volume is restored through abstinence or continues to decline with continued drinking remains unclear but probably involves changes in both myelination and axonal integrity.

Most early brain imaging studies of alcoholism were confined to male subjects. More recently, researchers have sought to determine whether women’s brains are more or less vulnerable than men’s to the damaging effects of alcohol abuse or dependence. A neuroimaging study that used computerized tomography (CT) showed comparable deficits in men and women, even though the women drank much less alcohol than the men (Jacobson 1986). This finding, which suggested that women were more vulnerable to alcohol-induced brain damage, has been supported by some MRI studies (Hommer et al. 2001) measuring volumes of cortical white and gray matter and the fluid that bathes the brain and spinal cord (cerebrospinal fluid [CSF]). Other studies have not supported the idea of increased vulnerability among women (Pfefferbaum et al. 2001). These later MRI studies highlight the importance of controlling adequately for gender-related differences in body/brain morphology and quantity and pattern of drinking. Furthermore, it is becoming increasingly apparent that brain tissue, especially white matter, that appears normal on MRI in alcoholic patients may in fact be affected by alcoholism.

## Diffusion Tensor Imaging

DTI shows particular promise for assessing white-matter damage that is associated with excessive alcohol use, as indicated by both post mortem and in vivo studies.

Conventional MR images are “pictures” primarily of free water, the concentration of which differs by tissue type: White matter consists of about 70 percent water, gray matter 80 percent, and CSF 99 percent. These differences in water content contribute to the contrast between tissue types visible on structural images. DTI takes this imaging further by measuring differences in the freedom with which water molecules move within a tissue type and the amount and orientation of their diffusion, especially in white matter. With appropriate data collection and processing techniques (Adalsteinsson et al. 2002), researchers can generate images that highlight white-matter tracts.

**Figure 1:**
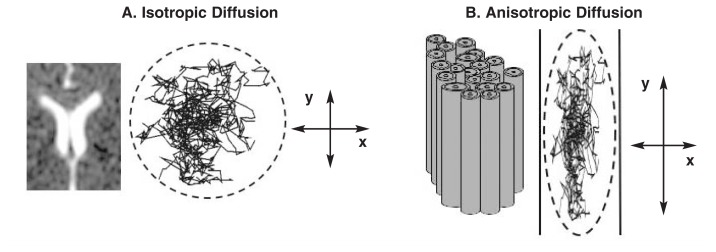
Isotropic and anisotropic diffusion. **(A)** Water molecules in the brain are constantly moving (i.e., in Brownian motion). When motion is unconstrained, as in the large fluid-filled spaces deep in the brain (i.e., the ventricles, as illustrated in the MR image on the left), diffusion is isotropic, which means that motion occurs equally and randomly in all directions. **(B)** When motion is constrained, as in white-matter tracts (illustrated on the right), diffusion is anisotropic, meaning that motion is oriented more in one direction than another (e.g., along the *y* axis rather than along the *x* axis).

Water molecules are in constant motion. In regions such as the ventricles, relatively large fluid-filled spaces deep in the brain, which offer few or no physical constraints, movement occurs randomly in every direction. This random movement is described as isotropic (*iso* meaning “same” and *tropic* meaning “movement”). By contrast, water molecules in white-matter fibers are constrained by the physical boundaries of the axon sheath, which cause greater movement along the long axis of the fiber than across it. This movement is called anisotropic (*aniso* meaning “not the same”) (see figure 1).

**Figure 2:**
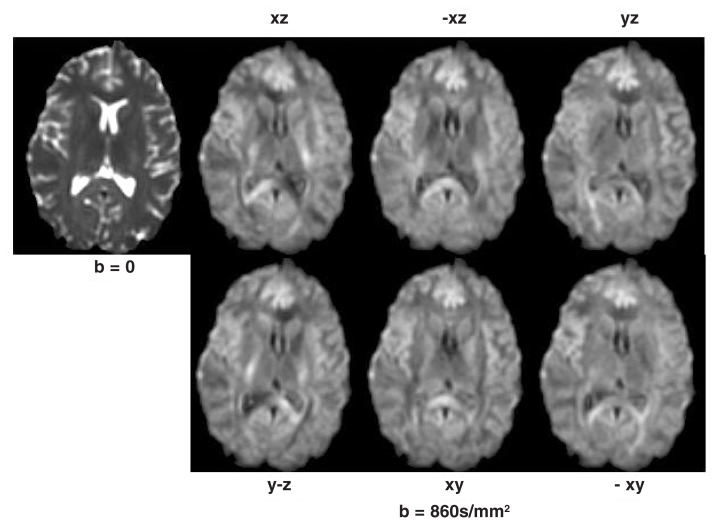
Images of the same axial slice of the brain acquired with varying diffusion gradients. The strength of the diffusion gradient is indicated by b. The image at the top left was acquired without diffusion gradients (b = 0). The remaining six images were acquired when applying diffusion gradients (b = 860s/mm2) in six of the many possible combinations of directions (i.e., x = left to right, −x = right to left, y = front to back, z = top to bottom, and −z = bottom to top). For example, in the bottom right picture, diffusion gradients were applied from right to left and from front to back. When no gradients were applied, regions such as the ventricles and sulci (spaces between folds of brain tissue), where there is free movement of molecules, appear bright. When gradients were applied, these spaces appear dark.

How do we detect water diffusion in the brain with imaging? One way is to apply extra magnetic field gradients (i.e., diffusion gradients) during image acquisition to yield what is called a diffusion-weighted image. This process is analogous to looking through a microscope, focusing on some relatively motionless solid structures and some particles that are in Brownian motion, unfocusing briefly, and then refocusing the microscope on the same location. The solid structures will come back into focus, but some of the freely moving particles that have moved will be out of focus. In the ventricles, molecules are free to move out of focus. In the white-matter tracts, where axon sheaths restrict movement to one primary direction, it is less likely that molecules will move out of focus (i.e., there is less diffusion). Unlike the microscope, which has only one focus/unfocus direction, the scanner can focus and refocus in multiple dimensions by applying diffusion gradients in different directions. The researcher collects one image without gradients and then six images, each with diffusion gradients applied in a different direction (see figure 2).

**Figure 3:**
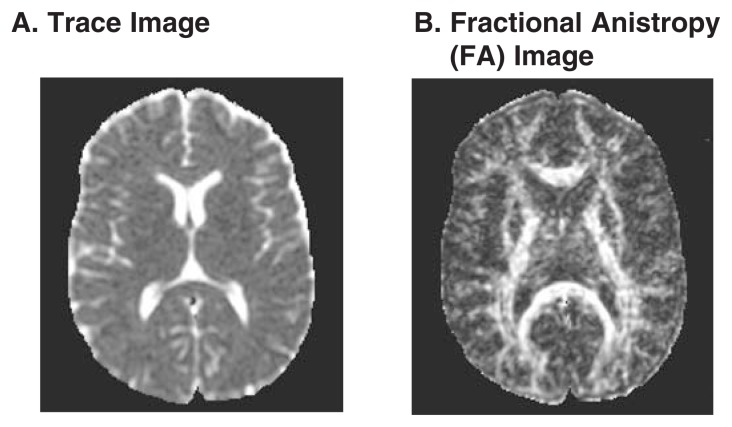
Two types of diffusion tensor imaging. **(A)** The trace image reflects the total amount of diffusion occurring in each region and highlights the ventricles, with little difference between white and gray matter. **(B)** The fractional anisotropy (FA) image highlights regions where diffusion is oriented in a single direction. The ventricles and gray matter are dark, whereas the white matter tracts are bright.

The technique is called diffusion *tensor* imaging because a tensor, a mathematical description of the orientation and magnitude of diffusion, is computed for each voxel from the seven images. Further calculations result in three summary measures that reflect the magnitude or amount of diffusion in each direction. Trace images are based on the average diffusion in all three directions and illustrate the overall magnitude of diffusion; in trace images, the ventricles, which contain few obstructions to movement, are bright, and the gray matter and white matter are both dark (see figure 3).

Fractional anisotropy (FA) images are based on the extent to which one direction dominates; they illustrate the degree to which water molecules move in one predominant orientation. If diffusion is unconstrained (i.e., isotropic), FA is close to zero. If diffusion has one primary orientation (i.e., is anisotropic), FA can approach 1. Because diffusion follows their orientation, the long, thin, cable-like bundles of fibers making up white-matter tracts appear bright on the FA image (see figure 3).

FA images acquired in different planes will highlight different white-matter tracts or provide different views of them (see the textbox, “Viewing the Brain”). Within a uniformly oriented tract, anything that disrupts the regular structure of white matter, such as loss of the protective myelin sheath or deterioration of the axons, might allow the water molecules to move more freely, resulting in decreased FA. Thus, lower-than-expected FA (which would indicate more isotropic diffusion) in a white-matter region of normal volume may reflect the loss of white-matter integrity resulting from a number of conditions, including aging and alcoholism.

**Viewing the Brain**

Fractional anisotropy (FA) images from a 31-year-old healthy man, showing upper (axial), middle (coronal), and lower (sagittal) orientations. Regions of higher intensity represent white-matter tracts. Examples of white-matter tracts are labeled. Areas where multiple white-matter tracts cross in different orientations, such as adjacent to the genu of the corpus callosum on axial view, appear lower in intensity because no single orientation predominates.

**Figure:**
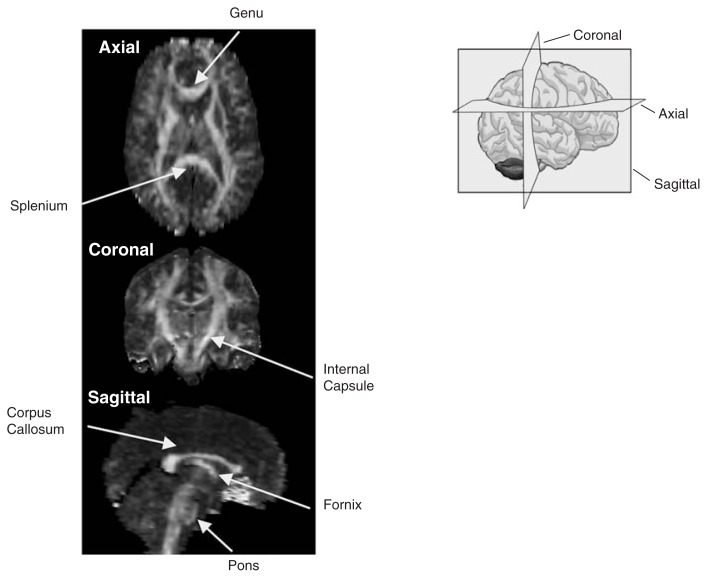

**Axial**: sliced along the horizontal plane

**Coronal**: sliced vertically, looking at the brain from the front or back

**Sagittal**: sliced vertically, looking at the brain from the side

**Genu**: front region of the corpus callosum

**Splenium**: back region of the corpus callosum

**Internal capsule**: the major route by which the cerebral cortex is connected with the brain stem and spinal cord

**Corpus callosum**: a band of white-matter fibers linking the brain’s hemispheres

**Fornix**: a pathway that carries information in the brain between the hippocampus and the mamillary bodies

**Pons**: a largely white-matter structure of the brain stem

**Figure 4:**
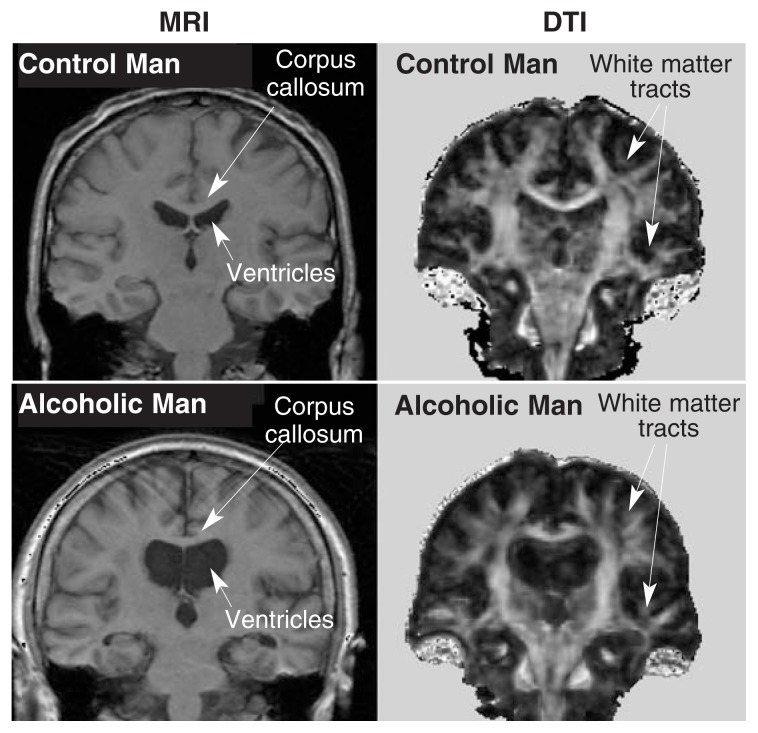
Images displayed in the coronal orientation from MRI and DTI studies of a 61-year-old healthy man (upper images) and a 60-year-old alcoholic man (lower images). The high-resolution MRI slices are at the same locations as the fractional anisotropy images of the DTI panels. Note on the MRI the thinner corpus callosum displaced upward by enlarged ventricles and, on the DTI, less well delineated white matter tracts in the alcoholic man compared with the healthy man.

In order to link FA values to specific white-matter structures and regions, the FA image should be aligned with an independently collected high-resolution structural MR image that provides the template for defining the white-matter regions of interest (see figure 4). This is the best approach to confirm that the FA values are in fact located in white-matter regions and requires special techniques to ensure accurate and valid matching of FA and structural images (Pfefferbaum et al. 2000*b*, Pfefferbaum and Sullivan 2003).

Another measure that can be computed with DTI is intervoxel (i.e., between voxels) rather than intravoxel (i.e., within voxel) coherence. This is the degree to which diffusion in neighboring voxels has a common orientation (Pfefferbaum et al. 2000*b*). This measure is similar to FA but views coherence on a larger spatial, voxel-to-voxel scale (in contrast with FA’s intravoxel scale).

## Application to Alcoholism

Although DTI has revealed white-matter abnormalities in certain neuropsychiatric conditions such as Alzheimer’s disease, schizophrenia, AIDS, and depression, as well as in normal aging (for review, see Sullivan and Pfefferbaum 2003), DTI has only recently been used to examine brain white-matter microstructural integrity in alcoholism. Selective white-matter degradation associated with alcoholism is well established from neuropathological reports (e.g., Harper and Kril 1990). DTI offers an especially relevant and safe imaging method to track the condition of white-matter microstructure over the course of alcoholism.

**Figure 5:**
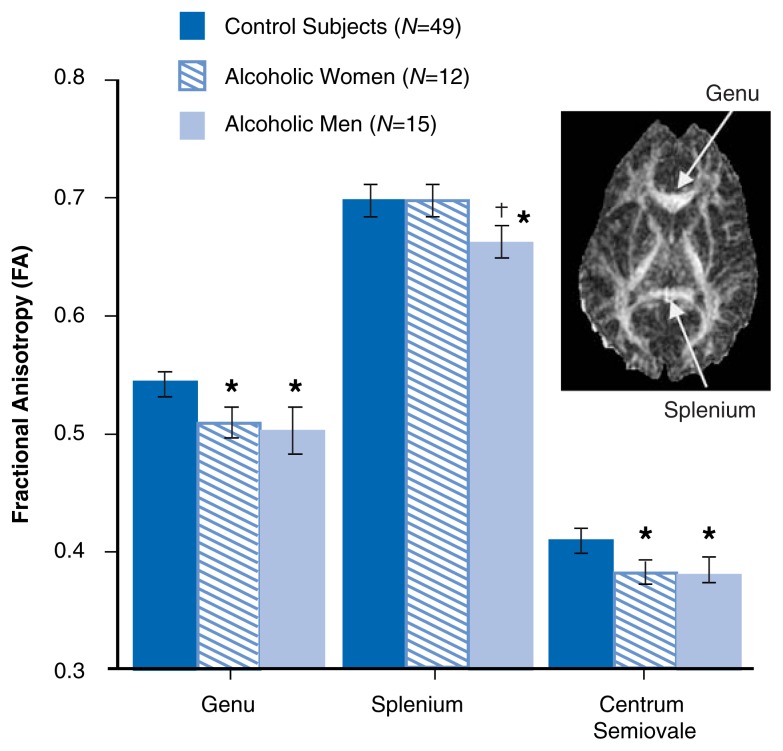
The colored bars represent the means, and the represents the standard errors of fractional anisotropy (FA) in three white-matter brain regions in 15 alcoholic men, 12 alcoholic women, and 49 healthy control men and women. As indicated by the stars, the alcoholic men and women had lower regional FA in the genu of the corpus callosum and the centrum semiovale (the mass of white matter composing the interior of the cerbral hemispheres). Only the alcoholic men had lower FA than control subjects in the splenium, as noted by the cross. SOURCES: Pfefferbaum et al. 2000a; Pfefferbaum and Sullivan 2002.

Research using DTI shows that, relative to age-matched control subjects, alcoholic men have lower regional FA, meaning that diffusion is less oriented in a single direction, in the front part (genu) of the corpus callosum and in the mass of white matter that composes the interior of the cerebral hemisphere (the side of the cerebrum; i.e., the centrum semiovale) (Pfefferbaum et al. 2000*a*) (see figure 5).

These DTI-detected white-matter abnormalities were functionally relevant; working memory correlated positively with FA in the white-matter region in the back part of the corpus callosum (i.e., the splenium), whereas attention scores correlated positively with intravoxel coherence in the genu (Pfefferbaum et al. 2000*a*). A study of alcoholic women revealed regional abnormalities in white-matter microstructure (see figure 5) not detectable with MRI macrostructural measures of size (Pfefferbaum et al. 2002; Pfefferbaum and Sullivan 2002). These results provide in vivo evidence that alcoholism disrupts white-matter microstructure and suggest that the interruption of both intra- and intervoxel coherence contributes to deficits in attention and working memory associated with chronic alcoholism. It remains to be determined whether DTI measures of white-matter integrity parallel the increase in white-matter volume that has been associated with maintaining abstinence (Shear et al. 1994) or the further decrease of white-matter volume associated with relapse after detoxification (Pfefferbaum et al. 1995).

## Conclusion

Conventional MRI and DTI modalities each quantify different aspects of brain macrostructure and microstructure. When used together to assess patients when they first stop chronic heavy drinking, and again after longer periods of sobriety or possible relapse, MRI and DTI represent a powerful means of characterizing brain changes at different stages of alcoholism. The different types of information provided can be used to test hypotheses about the factors underlying improvement with prolonged abstinence from alcohol or deterioration with resumption of drinking. For example, low levels of FA in white matter may signify reversible demyelination and axonal deterioration or permanent axonal degeneration. If retesting shows an increase in FA (i.e. increased orientation in one direction), this may suggest remyelination or regrowth of neuronal processes.

Behavioral studies could include tests that assess functions of cortical regions connected by the white-matter pathways found to be disrupted by alcoholism and then improved with abstinence. Patterns of recovery and deterioration derived from such in vivo neuroimaging studies may provide clues to cellular mechanisms underlying reversible and permanent brain structural and functional changes occurring during the course of alcoholism.

## References

[b1-146-152] Adalsteinsson E, Sullivan EV, Pfefferbaum A, Liu Y, Lovinger DM (2002). Biochemical, functional and microstructural magnetic resonance imaging (MRI). Methods in Alcohol-Related Neuroscience Research.

[b2-146-152] Charness ME (1993). Brain lesions in alcoholics. Alcoholism: Clinical and Experimental Research.

[b3-146-152] Harper CG, Kril JJ (1990). Neuropathology of alcoholism. Alcohol and Alcoholism.

[b4-146-152] Hommer D, Momenan R, Rawlings R (1996). Decreased corpus callosum size among alcoholic women. Archives of Neurology.

[b5-146-152] Hommer DW, Momenan R, Kaiser E, Rawlings RR (2001). Evidence for a gender-related effect of alcoholism on brain volumes. American Journal of Psychiatry.

[b6-146-152] Jacobson R (1986). The contributions of sex and drinking history to the CT brain scan changes in alcoholics. Psychological Medicine.

[b7-146-152] Jernigan TL, Butters N, DiTraglia G (1991). Reduced cerebral grey matter observed in alcoholics using magnetic resonance imaging. Alcoholism: Clinical and Experimental Research.

[b8-146-152] Oscar-Berman M, Noronha A, Eckardt M, Warren K (2000). Neuropsychological vulnerabilities in chronic alcoholism. Review of NIAAA’s Neuroscience and Behavioral Research Portfolio.

[b9-146-152] Pfefferbaum A, Sullivan EV (2002). Microstructural but not macrostructural disruption of white matter in women with chronic alcoholism. Neuroimage.

[b10-146-152] Pfefferbaum A, Sullivan EV (2003). Increased brain white matter diffusivity in normal adult aging: Relationship to anisotropy and partial voluming. Magnetic Resonance in Medicine.

[b11-146-152] Pfefferbaum A, Lim KO, Zipursky RB (1992). Brain gray and white matter volume loss accelerates with aging in chronic alcoholics: A quantitative MRI study. Alcoholism: Clinical and Experimental Research.

[b12-146-152] Pfefferbaum A, Sullivan EV, Mathalon DH (1995). Longitudinal changes in magnetic resonance imaging brain volumes in abstinent and relapsed alcoholics. Alcoholism: Clinical and Experimental Research.

[b13-146-152] Pfefferbaum A, Lim KO, Desmond J, Sullivan EV (1996). Thinning of the corpus callosum in older alcoholic men: A magnetic resonance imaging study. Alcoholism: Clinical and Experimental Research.

[b14-146-152] Pfefferbaum A, Sullivan EV, Mathalon DH, Lim KO (1997). Frontal lobe volume loss observed with magnetic resonance imaging in older chronic alcoholics. Alcoholism: Clinical and Experimental Research.

[b15-146-152] Pfefferbaum A, Sullivan EV, Rosenbloom MJ (1998). A controlled study of cortical gray matter and ventricular changes in alcoholic men over a five-year interval. Archives of General Psychiatry.

[b16-146-152] Pfefferbaum A, Sullivan EV, Hedehus M (2000a). In vivo detection and functional correlates of white matter microstructural disruption in chronic alcoholism. Alcoholism: Clinical and Experimental Research.

[b17-146-152] Pfefferbaum A, Sullivan EV, Hedehus M (2000b). Age-related decline in brain white matter anisotropy measured with spatially corrected echo-planar diffusion tensor imaging. Magnetic Resonance in Medicine.

[b18-146-152] Pfefferbaum A, Rosenbloom MJ, Deshmukh A, Sullivan EV (2001). Sex differences in the effects of alcohol on brain structure. American Journal of Psychiatry.

[b19-146-152] Pfefferbaum A, Rosenbloom MJ, Serventi K, Sullivan EV (2002). Corpus callosum, pons and cortical white matter in alcoholic women. Alcoholism: Clinical and Experimental Research.

[b20-146-152] Shear PK, Jernigan TL, Butters N (1994). Volumetric magnetic resonance imaging quantification of longitudinal brain changes in abstinent alcoholics. Alcoholism: Clinical and Experimental Research.

[b21-146-152] Sullivan EV, Noronha A, Eckardt M, Warren K (2000). Human brain vulnerability to alcoholism: Evidence from neuroimaging studies. Review of NIAAA’s Neuroscience and Behavioral Research Portfolio.

[b22-146-152] Sullivan EV, Pfefferbaum A (2003). Diffusion tensor imaging in normal aging and neuropsychiatric disorders. European Journal of Radiology.

[b23-146-152] Sullivan EV, Marsh L, Mathalon DH (1996). Relationship between alcohol withdrawal seizures and temporal lobe white matter volume deficits. Alcoholism: Clinical and Experimental Research.

[b24-146-152] Sullivan EV, Deshmukh A, Desmond JE (2000). Cerebellar volume decline in normal aging, alcoholism, and Korsakoff’s syndrome: Relation to ataxia. Neuropsychology.

[b25-146-152] Sullivan EV, Rosenbloom MJ, Serventi KL (2003). The effects of alcoholism–schizophrenia comorbidity on volumes of the thalamus and pons. American Journal of Psychiatry.

